# Population dynamics and threats to an apex predator outside protected areas: implications for carnivore management

**DOI:** 10.1098/rsos.161090

**Published:** 2017-04-19

**Authors:** Samual T. Williams, Kathryn S. Williams, Bradley P. Lewis, Russell A. Hill

**Affiliations:** 1 Department of Anthropology, Durham University, Dawson Building, South Road, Durham DH1 3LE, UK; 2 Primate and Predator Project, PO Box 522, Louis Trichardt, 0920, South Africa; 3 Department of Zoology, University of Venda, Private bag X5050, Thohoyandou, 0950, South Africa; 4 Bainbridge Island School District, 8489 Madison Avenue NE, Bainbridge Island, WA 98110, USA

**Keywords:** camera trap, telemetry, snaring, human–wildlife conflict, *Panthera pardus*, felid

## Abstract

Data on the population dynamics and threats to large carnivores are vital to conservation efforts, but these are hampered by a paucity of studies. For some species, such as the leopard (*Panthera pardus*), there is such uncertainty in population trends that leopard trophy hunting has been banned in South Africa since 2016 while further data on leopard abundance are collected. We present one of the first assessments of leopard population dynamics, and identify the key threats to a population of leopards outside of protected areas in South Africa. We conducted a long-term trap survey between 2012 and 2016 in the Soutpansberg Mountains, and drew on a previous estimate of leopard population density for the region from 2008. In 24 sampling periods, we estimated the population density and assessed population structure. We fitted eight leopards with GPS collars to assess threats to the population. Leopard population density declined by 66%, from 10.73 to 3.65 leopards per 100 km^2^ in 2008 and 2016, respectively. Collared leopards had a high mortality rate, which appeared to be due to illegal human activity. While improving the management of trophy hunting is important, we suggest that mitigating human–wildlife conflict could have a bigger impact on carnivore conservation.

## 1. Introduction

Large mammalian carnivores are incredibly important to ecosystems and environments. As apex predators, the extirpation of carnivores can trigger trophic cascades that can reduce biodiversity [1], increase the transmission of infectious diseases to humans [2], increase crop damage [3], reduce carbon sequestration [4] and even modify river morphology [5]. They are among the most sought after species by tourists [6,7], and they are of great economic significance through the tourism and hunting industries [8,9]. Carnivores are also incredibly important to human societies. People tell stories about large carnivores in traditional fables, their likenesses inspire artwork, they play roles in witchcraft, and their products are used in traditional rituals and medicine [10]. But despite their value, 59% of large carnivores are now threatened with extinction [11], and this will be exacerbated as humans continue to modify the environment [12]. Carnivores frequently come into conflict with humans [13,14], and anthropogenic threats such as persecution [15], loss of habitat and decline in prey base [16] have led to massive population declines and range contractions for most large carnivores [17].

On average, large carnivore species have lost 53% of their historic range [17]. For some species, this range loss has been much greater, with the leopard (*Panthera pardus*) having lost 63–75% of its historic range worldwide [18] and 80% of its past range in South Africa [19], the most extensive decline in southern Africa [18]. As a consequence, the leopard has recently been uplisted to Vulnerable on both the global IUCN Red List [20] and the Red List of Mammals of South Africa, Swaziland and Lesotho [21], highlighting an increasing concern over its conservation status.

Although it is clear that the range of leopards is contracting, there is a dearth of long-term data on population size and threats faced by leopards [18]. Insufficient data also hinders the management of other carnivores such as the brown hyaena (*Hyaena brunnea*) [22] and black-footed cat (*Felis nigripes*) [23]. In South Africa, there is an urgent need to determine the population trends of leopards to inform leopard management [24], and there is such uncertainty about the abundance of leopards that leopard trophy hunting has been banned in South Africa since 2016 while robust data are sought in order to allow hunting quotas to be set at sustainable levels [18]. This is especially pertinent given the high degree of public scrutiny on trophy hunting of large carnivores in Africa [25] in the wake of the recent controversial hunt of Cecil the lion (*Panthera leo*) in Zimbabwe [26]. Research that assists leopard management is considered a priority [27], such as determining the population density and trends, demography and identifying any threats to local leopard populations [24,28]. Such information about local leopard populations, which can be defined as a group of individuals within investigator-delimited areas [29], is vital to leopard management and conservation efforts [28].

Assessing leopard population trends, demography and threats is particularly important outside of state-protected areas, such as on private land. In South Africa, 68% of remaining leopard habitat is outside of legally protected areas [19], and leopard conservation efforts should be focused outside of protected areas [19], where leopards are most at risk [30]. Furthermore, leopard management strategies are focused on regulating legal and illegal utilization of leopards, most of which occur outside protected areas. One area likely to be of great importance to leopard conservation is the Soutpansberg Mountains in South Africa, of which very little is formally protected [31]. The Soutpansberg Mountains are a biodiversity hotspot [32], supporting high species diversity [33,34] including in 2008 one of the highest reported densities of leopards in Africa [35]. The current population density, population trends, changes in leopard demography and threats to this leopard population are, however, unknown. We present the first estimates of trends in leopard population density and abundance in the Soutpansberg Mountains as a case study to inform future research and management focusing on large carnivore population dynamics. We also assess changes in the demographics of the population, and identify key threats.

## 2. Material and methods

### 2.1. Study area

The study was conducted in the western Soutpansberg Mountains, Limpopo Province, South Africa. The mountains cover an area of 6800 km^2^ [34] and study site (central coordinates S29.44031°, E23.02217°) elevation varies from 750 to 1748 m.a.s.l. [36]. Climate is characterized by a warm, wet season (October to March) and a cool, dry season (April to September) [37]. Land uses include a private nature reserve, ecotourism, hunting, and farming of livestock, game and crops. Most of the land is privately owned, but community owned land was also present within the area.

### 2.2. Spatially explicit capture recapture

**Figure 1.**
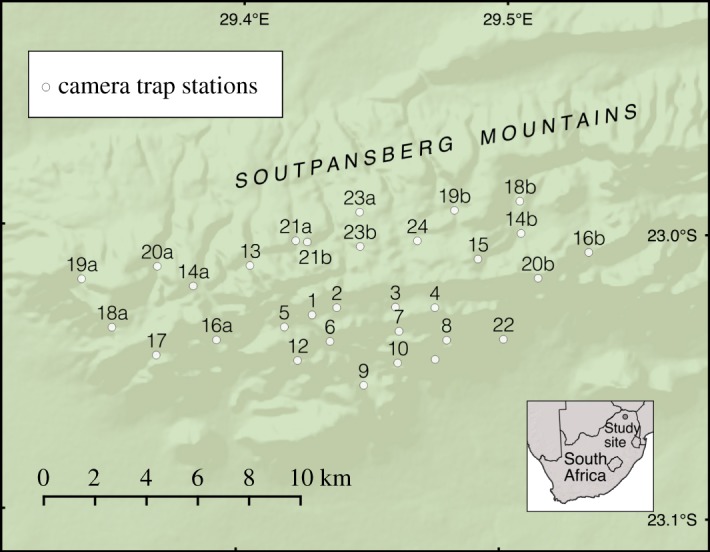
Locations of the camera traps for the survey of leopard population density and demography in the Soutpansberg Mountains.

An array of Reconyx Hyperfire HC500 and HC600 camera traps was established to estimate trends in leopard population density and demography. Forty-six camera traps were placed in pairs at 23 camera trap stations across the study site, encompassing the study region surveyed in 2008 [35]. Nineteen camera trap stations remained in the same locations throughout study, but five stations were relocated due to the withdrawal of one landowner in 2013 (figure 1; electronic supplementary material, table A1), reducing the area covered by the cameras from 73 to 59 km^2^. Camera trap stations were situated on roads, drainage lines and game trails where leopard signs had been recorded, to maximize the probability of photographing leopards. A maximum spacing of 3 km between camera traps stations was used [35] to ensure that there were no gaps in the array large enough to encompass the entire home range of an adult female leopard (20 km^2^ at this study site [21]), so that all individuals had a capture probability greater than zero [38,39]. The home range size of adult female leopards was selected as they tend to have smaller ranges than adult males [40], so are more likely to have a capture probability of zero. Camera traps were mounted on trees or poles approximately 40 cm above the ground. The cameras ran continuously between 01 January 2012 and 02 February 2016 (electronic supplementary material, table A2), with the minimum delay between captures (approx. 1 s). Each camera trap was visited every two to four weeks to change batteries, ensure that the cameras remained operational, and to download the photographs. Individual leopards were identified from photographs using their unique coat pattern, and were allocated into adult male, adult female and sub-adult categories using body size, the appearance of external genitalia, and secondary sexual characteristics such as build and the dewlap [41]. Adults were defined as at least 2 years old, and sub-adults were excluded from density estimation [35].

### 2.3. Individual monitoring

Threats to the leopard population were assessed by determining the fate of collared animals. Leopards were captured using soft-hold foot loops [42], and immobilized by a South African Veterinary Council-registered veterinarian using Zoletil, or a combination of Zoletil and Medetomidine delivered using a Dan-inject CO_2_ rifle. Medetomidine was reversed using Atipamezole. Vectronic GPS Plus collars were fitted to a total of eight adult leopards (electronic supplementary material, table A3). The collars recorded the coordinates of the study animals at 200-min intervals and were fitted with activity sensors that triggered a mortality signal when no movement was detected for 24 h. The data were transmitted to the users by UHF radio link, and also over the mobile GSM network by SMS, enabling the study animals to be located quickly after death to determine the cause. The collars were also fitted with VHF transmitters to enable locating of collared animals in real time. Collars were fitted with electronic drop-off devices by Vectronic that allowed the unit to disengage automatically after a specified duration. The disengagement date was set at 455 days after deployment, as by this stage the collar batteries would be almost depleted, but would still have sufficient power to facilitate retrieval of the collar.

### 2.4. Statistical analysis

Camera trap data were analysed following Chase Grey *et al.* [35] in order to enable comparisons. Data on the locations and trapping occasions on which individual leopards were captured were used to create a spatially explicit capture–recapture model [43] employing a Bayesian framework [44] to estimate leopard density using SPACECAP v. 1.0.1 [45] in R v. 3.3.0 [46]. The duration of each trapping occasion was set at 24 h and the dataset was divided into 24 sampling periods, each lasting 60 days, with each sampling period separated by 1 day (electronic supplementary material, table A2). A state space pixel size of 0.25 km^2^ was used, and a buffer of 20 km around the camera traps was employed to encompass the home ranges of all leopards that were photographed [47]. Potential home range centres were scored as unsuitable habitat when they overlapped with urban areas. Spatial capture–recapture models were constructed using the Bernoulli encounter process and half normal detection function. Between 100 000 and 200 000 iterations were used in the Markov-chain Monte Carlo simulation, along with a burn-in of 50 000–80 000 iterations, a thinning rate of 5–10, and data augmentation of 200–700 individuals. Model parameters were adjusted until convergence was good (Geweke *z*-scores were between −1.6 and 1.6) [48], Bayesian *p*-values did not approach 0 or 1 [49], and data augmentation, state space extent and sample size were sufficiently large (see the electronic supplementary material, table A2) [45]. Population structure was assessed by summing the number of unique individuals of each age sex class photographed in each sampling period. Trends in population density and demography were analysed using linear regression, and Wilcoxon rank sum tests were used to assess differences in the number of sampling periods for which adult males and adult females remained present. Statistical analysis was conducted using R v. 3.3.0 [46]. All data underlying the analyses are publically available [50].

## 3. Results

### 3.1. Population dynamics

**Figure 2.**
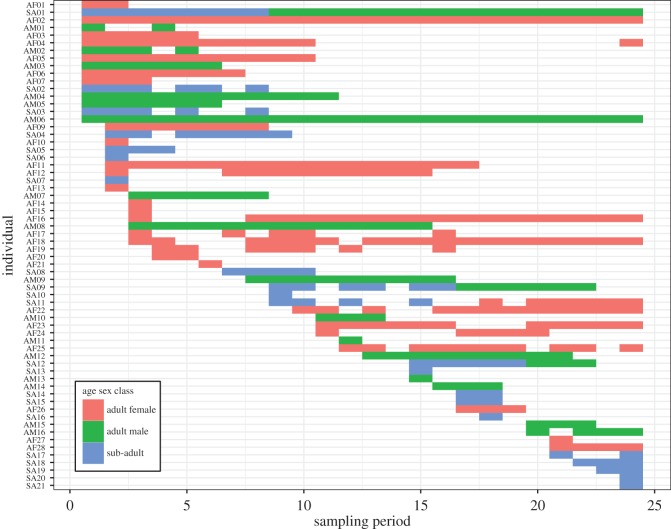
Sampling periods in which individual leopards were photographed on camera traps in the Soutpansberg Mountains between 2012 and 2016 (see the electronic supplementary material, table A2 for dates).

**Figure 3.**
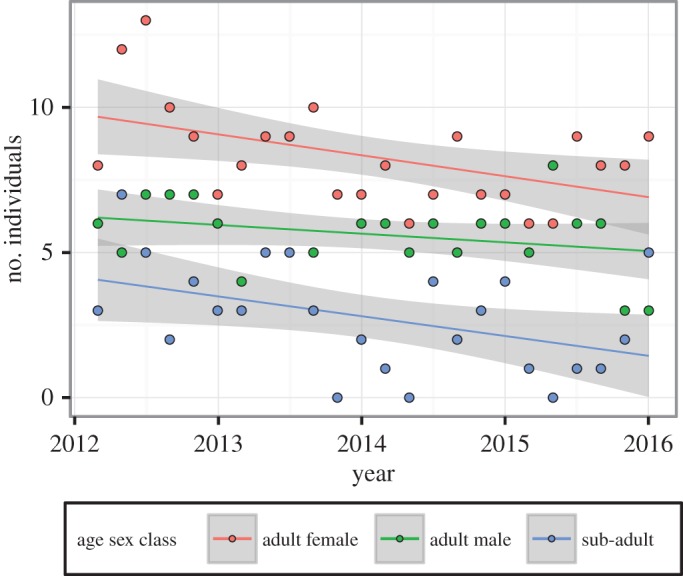
Change in the number of individual leopards identified per sampling period in each age sex class in the Soutpansberg Mountains between 2012 and 2016. Shading represents 95% CIs.

A total of 16 adult male leopards and 28 adult females were photographed. Twenty-one sub-adults were also recorded, of which three became adult males and one an adult female during the course of the study. The mean adult male to adult female sex ratio was 1 : 1.65. The tenure of each individual leopard is shown in figure 2. There was no difference in the number of sampling periods for which adult males and adult females remained present (*W* = 276.5, d.f. = 1, *p* = 0.5276). The number of adult males identified in each study period remained stable, while the number of adult females, sub-adults and total number of leopards declined significantly between 2012 and 2016 (figure 3 and table 1).

**Table 1.** Results of linear regression of the number of individual leopards identified per sampling period against date.

	model		coefficients			
age sex class	*F*_1,22_	*R*^2^	estimate	s.e.	*t*	*p*-value
adult male	2.057	0.0855	−0.0008	0.0006	−1.434	0.1656
adult female	6.761	0.2351	−0.0019	0.0007	−2.600	0.0163
sub-adult	5.006	0.1854	−0.0019	0.0008	−2.237	0.0357
all leopards	13.250	0.3759	−0.0047	0.0013	−3.640	0.0014

**Figure 4.**
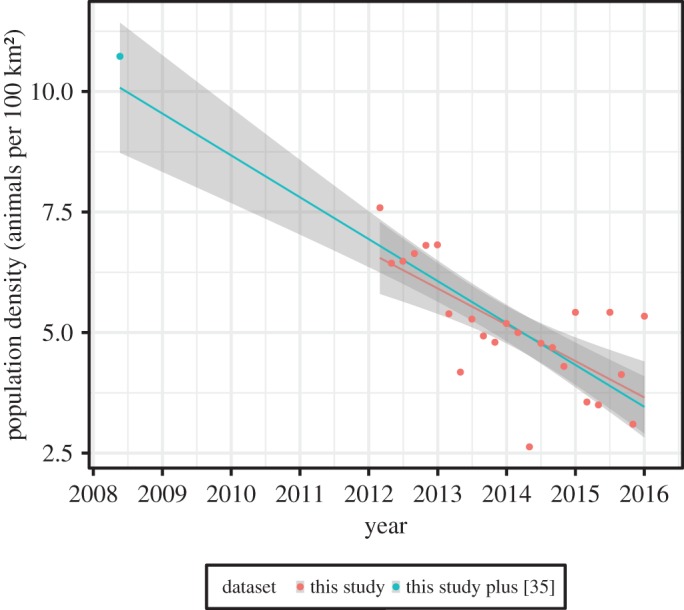
Change in the population density of leopards in the Soutpansberg Mountains between 2008 and 2016. Shading represents 95% CIs.

Leopard density in the Soutpansberg ranged from 6.55 in 2012 to 3.65 in 2016 (electronic supplementary material, table A2). Leopard density declined linearly across the study (*F*_1,22_ = 22.04, *p* = 0.0001, *R*^2^ = 0.5005; figure 4) with a decline of 44% over approximately 4 years (a reduction of 0.75 leopards per 100 km^2^ every year). Incorporating the density estimate available in Chase Grey *et al.* [35] produced a similar model (*F*_1,23_ = 57.66, *p* < 0.0001, *R*^2^ = 0.7149; figure 4), but indicated a 66% decline over a period of just over seven and a half years (0.87 leopards per 100 km^2^ per year).

### 3.2. Individual monitoring

Only two of eight leopards collared (25.0%) survived to the end of the 455-day collaring period. Three were killed by snares (37.5%) and one was shot without a permit for perceived cattle predation (12.5%). Two collared leopards went missing (25.0%), suspected dead, since they disappeared from the camera trap photographs at the same time.

## 4. Discussion

Leopard population density in the western Soutpansberg in 2012 (6.55 animals per 100 km^2^) was similar to published values at other sites [51–53], but by 2016 had dropped substantially to 3.65 animals per 100 km^2^. This also contrasts with the relatively high densities reported for the area in 2008 [35]. The density of leopards in the Soutpansberg Mountains has decreased by 44% since 2012 and by 66% since 2008, an extremely rapid decline. If this trend continues at the same rate, the population will essentially disappear from the Soutpansberg Mountains before 2020. Owing to the topography of the mountains the western Soutpansberg leopard population has relatively hard boundaries, being surrounded by human-dominated farming landscapes. This has been identified as an area of sub-optimal connectivity of suitable leopard habitat [54]. As a result, the local population is relatively isolated from immigration, making it particularly sensitive to mortalities, as these are likely to be relatively rarely compensated by immigration.

There are very few other studies of population trends of leopards with which to compare this decline [18,24]. The only other comparable study estimated a 56% increase in the density of leopards in Phinda Private Game Reserve, South Africa over 4 years due to management interventions [55]. Nevertheless, studies on lions [56] and on black-backed jackals (*Canis mesomelas*) and bat-eared foxes (*Otocyon megalotis*) [57] have noted similar trends where populations have been monitored over multiple years, suggesting the declines in the density of African carnivores exemplify global trends [17].

The decline observed in leopard density appeared to be driven by a decrease in the number of adult females, while the number of adult males was more stable. Although sub-adults were excluded from the density estimates, the number of sub-adults photographed also declined over the course of the study, most probably linked to the decline in adult females photographed. Low sub-adult survival rates would reinforce the population decline, making a future recovery less likely without compensatory immigration. The reasons why the number of males photographed per sampling period remained stable while the number of females and sub-adults photographed was declining is not clear, as we did not expect the main threats to the population to affect adult males less than adult females. Declines in the number of adult males could have been masked by the maturation of sub-adults into adult males; during the course of the study three sub-adults matured into adult males, while only one sub-adult matured into an adult female. Furthermore, only three adult males were photographed in the final two sampling periods, the lowest level of all 24 sampling periods. Continued monitoring will allow determination of whether a negative trend in the number of adult males is also evident in the population. The lower reduction in males might also be buffered in the short term by their greater dispersal and ranging distances relative to females [40,58], as unoccupied areas may be more quickly located and filled by males than females in the population. A limitation of estimating leopard density using SPACECAP is that it is not currently possible to incorporate covariates such as sex. This would have been interesting, as the numbers of individual adult male and female leopards in the local population appeared to be changing at different rates.

Care must be taken when interpreting data on the threats that are driving these population declines due to the limited sample size of collared leopards. Nevertheless, the death of six of eight collared leopards over the 455-day period for which each collar was deployed suggested a very high rate of mortality. Death was the most likely explanation for the collared leopards that went missing, as we stopped receiving data from their collars at the same time as they stopped appearing in images on the camera traps. Emigration or collar failure is thus unlikely. Instead it is probable that they died either in an area where the signals would be obscured (such as in a cave), or the collars were destroyed deliberately, indicating anthropogenic mortality. Illegal hunting was the sole cause of the known deaths of collared leopards. Furthermore, local conservation actions are known to have prevented poisoning of one collared leopard following a livestock predation event, without which mortality rates would have been higher.

Our data indicate that illegal human activity could be the primary cause of leopard mortality in the study area, often in retaliation to perceived livestock predation or for bushmeat, and this may be driving steep declines in the leopard population. Anthropogenic mortality is often the biggest threat to leopards outside of protected areas [14], and similar results have been reported for other large carnivores [30,59,60]. The sex ratio was similar to other sites [51,61], and was not indicative of overexploitation of males through trophy hunting [62]. As reported in the Waterberg District Municipality in South Africa [63,64], legal mechanisms of leopard removal such as trophy hunting and damage causing animal removals appear to be less important threats to the leopard population than illegal activities such as snaring [14] in the Soutpansberg. Snaring can be a serious threat to large carnivores, and additional research is required to fully understand the impact of this on large felids [65].

In this case study, the leading causes of leopard mortality were snaring, shooting and poisoning, either in response to the perceived risk of livestock predation or poaching for bushmeat or animal parts. In this case, we thus recommend increasing efforts to engage with local communities to reduce the level of these activities, for example, through education [66] and enhancing livestock husbandry [67,68]. Efforts to reduce human–carnivore conflict can be very successful at promoting the recovery of leopard populations [55]. Further investigations, drawing on approaches from the social sciences [69], into the underlying causes of illegal activity leading to leopard mortality should also be undertaken in order to guide conservation actions. Effective strategies for managing damage-causing animals should also be developed and adhered to [24], such as the draft national norms and standards recently published for South Africa [70].

Although retaliatory killings may present the largest threats to leopard populations outside protected areas [64], this study calls into question the sustainability of additive off take through legal mechanisms of leopard removals such as trophy hunting and damage-causing animal destruction permits [71]. Furthermore, trophy hunting of large carnivores can be associated with elevated levels of human–wildlife conflict and increased mortality from persecution [72]. Declines in leopard density may also increase human–wildlife conflict through the mesopredator release effect [73] since caracals (*Caracal caracal*), black-backed jackals and baboons (*Papio ursinus*) are responsible for significant agricultural damage [74]. Since leopards cause less livestock damage than farmers perceive [75], this would result in elevated levels of livestock and crop damage and increased retaliatory killing.

In some cases, trophy hunting can be the main driver of population declines [55,59], and improved management of leopard trophy hunting is urgently required [24]. Before the national ban on trophy hunting leopards came into effect in 2016 [18] leopards were over-harvested in South Africa [76], which was partly responsible for predicted population declines [64]. An adaptive management system for managing leopards in South Africa is currently being developed, and much effort to date has centred on the regulation of leopard trophy hunting [77]. Under the new adaptive management system, before the trophy hunting ban female leopards were removed from hunting quotas and leopard hunting effort was spread across leopard hunting zones [76] in relation to leopard habitat suitability [19]. If the ban is lifted we recommend closure of the hunting zones [76] in which leopard populations are declining until the population has recovered. Such an approach will require intensive monitoring. In order to assess trends in carnivore population density, we advocate conducting multiple surveys over several years, as this mitigates the problem of variation in estimates and enables determination of population trends with a high degree of confidence. Few other studies have attempted this, with most calculating a single point estimate [35,51,52] rather than conducting multiple assessments over time (but see [55,57]).

## 5. Conclusion

The density of leopards in the case study declined by 66% over seven and a half years. The number of adult males was relatively stable, while the number of adult females and cubs declined over the course of the study. Illegal anthropogenic threats such as snaring, shooting and poisoning appear to be the main threats to the population. To date much attention has focused on improving trophy hunting of large carnivores, but our data suggest that the importance of other sources of anthropogenic mortality should not be overlooked, and efforts to mitigate these threats could have a bigger impact on the conservation status of large carnivores than improving legal trophy hunting.

## Supplementary Material

Table A1

## Supplementary Material

Table A2

## Supplementary Material

Table A3

## Supplementary Material

Afrikaans abstract

## Data accessibility

The data are deposited at Dryad: http://dx.doi.org/10.5061/dryad.n572r [50].
