# Supplementary material for: Population dynamics and threats to an apex predator outside protected areas: implications for carnivore management
Source: R Soc Open Sci. 2017 Apr 19;4(4):161090. doi: 10.1098/rsos.161090 (PMC5414262; doi:10.1098/rsos.161090)
Supplement: Afrikaans abstract [file rsos161090supp4.pdf]

Bevolkingsdinamika en bedreigings vir roofdiere aan die bopunt van die voedselketting buite beskermde gebiede: Implikasies vir die beheer van karnivore

Samual T. Williams, Kathryn S. Williams, Bradley P. Lewis, Russell A. Hill

Royal Society Open Science (2017)

Data oor die bevolkingsdinamika en bedreigings vir groot karnivore is noodsaaklik vir bewaringspogings, maar dit word in die wiele gery deur 'n gebrek aan studies. Vir sommige spesies, soos die luiperd (*Panthera pardus*), is daar so min inligting oor bevolkingstendense beskikbaar, dat luiperdtrofeëjag sedert 2016 in Suid-Afrika verban is, terwyl verdere inligting oor die luiperdgetalle ingesamel word. Ons bied een van die eerste studies oor die luiperdbevolking se dinamika en identifiseer die belangrikste bedreigings vir die luiperdbevolking buite beskermde gebiede in Suid-Afrika. Ons het 'n langtermyn kamerstrik-opname tussen 2012 en 2016 in die Soutpansberg gedoen en gebruik gemaak van 'n vorige opname oor die luiperd bevolkingsdigtheid vir die streek in 2008. Oor ‘n tydperk van 24 steekproewe het ons die bevolkingsdigtheid geskat en die bevolkingstruktuur geëvalueer. Ons het agt luiperds met GPS halsbande toegerus om bedreigings vir die luiperdbevolking te identifiseer. Die luiperds se bevolkingsdigtheid het met 66% afgeneem, van 10,73 tot 3,65 luiperds per 100 km² in 2008 en 2016 onderskeidelik. Daar is ‘n hoë sterftesyfer onder luiperds met GPS halsbande, wat die gevolg blyk te wees van onwettige menslike aktiwiteite.Terwyl die verbetering van die bestuur van trofeëjag belangrik is, stel ons voor dat verminderde mens-dier-konflik 'n groter impak op die bewaring van groot karnivore kan hê.

Vertaler: Annaline Smit

Note: Any differences in wording between the English and Afrikaans versions of the abstract do not affect the overall meaning.
